# Muscle sympathetic nerve activity‐coupled changes in brain activity during sustained muscle pain

**DOI:** 10.1002/brb3.888

**Published:** 2018-02-07

**Authors:** Sophie Kobuch, Azharuddin Fazalbhoy, Rachael Brown, Vaughan G. Macefield, Luke A. Henderson

**Affiliations:** ^1^ School of Medicine Western Sydney University Sydney NSW Australia; ^2^ Neuroscience Research Australia Sydney NSW Australia; ^3^ School of Health Sciences RMIT University Melbourne Vic. Australia; ^4^ College of Medicine Mohammed Bin Rashid University of Medicine & Health Sciences Dubai UAE; ^5^ Department of Anatomy and Histology University of Sydney Sydney NSW Australia

**Keywords:** midbrain periaqueductal gray, rostral ventrolateral medulla, sympathetic drive, tonic muscle pain

## Abstract

**Introduction:**

Long‐lasting experimental muscle pain elicits divergent muscle sympathetic responses, with some individuals exhibiting a persistent increase in muscle sympathetic nerve activity (MSNA), and others a decrease. These divergent responses are thought to result from sustained functional changes in specific brain regions that modulate the cardiovascular responses to pain.

**Aim:**

The aim of this study was to investigate brain regions that are functionally coupled to the generation of an MSNA burst at rest and to determine their behavior during tonic muscle pain.

**Methods:**

Functional magnetic resonance imaging of the brain was performed concurrently with microelectrode recording of MSNA from the common peroneal nerve during a 40 min infusion of hypertonic saline into the ipsilateral tibialis anterior muscle of 37 healthy human subjects.

**Results:**

At rest, blood oxygen level‐dependent signal intensity coupled to bursts of MSNA increased in the rostral ventrolateral medulla, insula, dorsolateral prefrontal cortex, posterior cingulate cortex, and precuneus and decreased in the region of the midbrain periaqueductal gray. During pain, MSNA‐coupled signal intensity was greater in the region of the nucleus tractus solitarius, midbrain periaqueductal gray, dorsolateral prefrontal, medial prefrontal, and anterior cingulate cortices, than at rest. Conversely, MSNA‐coupled signal intensity decreased during pain in parts of the prefrontal cortex.

**Conclusions:**

These results suggest that multiple brain regions are recruited in a burst‐to‐burst manner, and the magnitude of these signal changes is correlated to the overall change in MSNA amplitude during tonic muscle pain.

## 1 INTRODUCTION

Investigations in experimental animals have revealed that pain arising from the skin (cutaneous pain) evokes increases in blood pressure (BP), heart rate (HR), and sympathetic vasoconstrictor drive, whereas pain arising from muscle (deep pain) evokes decreases in these parameters (Keay & Bandler, 2001). These distinct autonomic responses are mediated by neurons in the midbrain periaqueductal gray matter (PAG), which are themselves under the influence of higher brain centers (Bandler, Keay, Floyd, & Price, 2000). In humans, the sympathetic vasoconstrictor drive is revealed through either skin or muscle sympathetic nerve activity (SSNA or MSNA), which can be recorded via microelectrodes inserted directly into a peripheral nerve in awake human subjects, a technique called microneurography. MSNA, which is tightly coupled to cardiac rhythmicity, reflects the activity of postganglionic sympathetic neurons supplying the skeletal muscle vascular beds. An increase in MSNA causes vasoconstriction and thereby increases BP (Macefield, 2013). While distinctive cardiovascular responses to pain originating from different tissues have been observed in humans (Feinstein, Langton, Jameson, & Schiller, 1954; Lewis, 1942), we recently reported that experimentally induced, transient (~6 min) cutaneous and muscle pain did not evoke such distinct cardiovascular responses. *Cutaneous* pain, elicited by subcutaneous injection of hypertonic saline solution, evoked increases in MSNA, BP, and HR. On the other hand, *muscle pain*, elicited by intramuscular injection—on average—also evoked significant increases in these cardiovascular parameters (Burton, Birznieks, Bolton, Henderson, & Macefield, 2009). We speculated at the time that this unexpected pattern of change during acute muscle pain may be a characteristic of the short lasting nature of the stimulus and the associated novelty.

We have since shown that prolonged (~45 min) muscle pain in humans evokes a mixed cardiovascular response, with some individuals showing a sustained MSNA increase, and others a sustained decrease (Fazalbhoy, Birznieks, & Macefield, 2012, 2014; Kobuch, Fazalbhoy, Brown, Henderson, & Macefield, 2017; Kobuch, Fazalbhoy, Brown, & Macefield, 2015, 2016). These individual differences are reliable across multiple experimental sessions in individuals (Fazalbhoy et al., 2014) and are not influenced by sex, age, anxiety levels, attitudes to pain, or resting MSNA, BP, or HR levels (Kobuch et al., 2015, 2016). Using concurrent functional magnetic resonance imaging (fMRI) and microneurography, we recently found that areas of the prefrontal, cingulate and precuneus cortices, hypothalamus, midbrain, and medulla displayed sustained increases in BOLD (blood oxygen level dependent) signal intensity in those individuals who displayed sustained increases in MSNA compared with those who displayed sustained decreases (Kobuch et al., 2017). That study explored continuous signal intensity changes throughout the whole scanning period, with subjects separated into two groups according to whether they exhibited an increase or a decrease in MSNA during pain (Kobuch et al., 2017). However, while investigating overall signal intensity changes associated with sustained increases or decreases in MSNA responses during tonic muscle pain, it did not explore MSNA‐coupled changes in BOLD signal intensity, that is, regions in which signal intensity increased or decreased during each MSNA burst. This is important, as sustained signal changes may be providing persistent modulatory inputs onto other brain regions that are actually driving each MSNA burst.

For example, at rest, signal intensity within the prefrontal, insular and precuneus cortices, hypothalamus, and medulla are tightly coupled to each MSNA burst (see Macefield & Henderson, 2016 for review). It is unknown whether, during sustained muscle pain, signal intensity within these areas remains coupled to MSNA, and/or whether other brain regions react in a similar manner. This is an important question as it is not known whether, in humans, there is an invariant set of brain regions in which activity is coupled to MSNA or whether other brain regions are recruited during various autonomic challenges.

The aim of this study was to identify brain regions that are functionally coupled to the generation of bursts of MSNA, both at rest and during tonic muscle pain. We hypothesized that, in addition to areas shown to be coupled to MSNA bursts at rest, additional regions, such as the cingulate cortex and midbrain, would also exhibit coupling to MSNA during pain and that increases in BOLD signal intensity in these areas would parallel an increase in MSNA burst amplitude.

## 2 METHODS

### 2.1 Participants

Experiments were performed on 37 healthy subjects (11 females; mean ± *SEM* age: 21.9 ± .5 years, range: 18–31 years). The subjects were recruited through an advertising flyer at the Western Sydney University School of Medicine. Exclusion criteria included a history of cardiovascular disease or chronic musculoskeletal pain. The subjects who volunteered for the experiment did so knowing that they were going to have a microelectrode inserted into a nerve, another needle inserted under the skin, and a cannula inserted into a muscle. Moreover, they knew they were going to experience strong muscle pain during the intramuscular infusion of hypertonic saline for up to an hour. All experiments were conducted at 2 p.m., and the subjects were instructed to abstain from any strenuous exercise and from drinking caffeine. None of the subjects took any medication for cardiovascular disease or pain relief. Most participants were part of a larger study (Kobuch et al., 2016) that included the completion of the following questionnaires: Pain Catastrophizing Scale (PCS) (Sullivan, Bishop, & Pivik, 1995), Pain Vigilance and Awareness Questionnaire (PVAQ) (McCracken, 1997), Pain Anxiety Symptoms Scale (PASS‐20) (McCracken & Dhingra, 2002), and the State and Trait Anxiety Inventory (STAI) (Spielberger, Gorsuch, Lushene, Vagg, & Jacobs, 1983).

All procedures were approved by the Human Research Ethics Committees of Western Sydney University and the University of New South Wales. Written consent was obtained from all subjects in accordance with the Declaration of Helsinki.

### 2.2 Microneurography

Subjects lay supine on a magnetic resonance imaging (MRI) bed with the legs supported by a foam cushion. Prior to entering the scanning room, a tungsten microelectrode was inserted percutaneously into a muscle fascicle of the right common peroneal nerve. The course of the right common peroneal nerve was identified via external stimulation (2–10 mA) using a 1‐mm surface probe that delivered .2 ms pulses at 1 Hz from an isolated constant‐current stimulator (Stimulus Isolator; ADInstruments, Sydney, Australia). Once twitches were evoked, the skin was marked over the optimal site for generating a twitch. This allowed the localization of the common peroneal nerve and the site for percutaneous insertion of tungsten microelectrodes (FHC, Bowdoin, ME, USA). In order to locate a specific nerve fascicle, intraneural stimulation through the microelectrode, with a maximal current of 1 mA at 1 Hz, was used to generate muscle twitches while adjusting the depth and angle of the microelectrode to optimize the responses. As the microelectrode approached the fascicle, the current was progressively reduced; twitches at currents of 0.02 mA or lower indicated that the microelectrode tip had entered a muscle fascicle. Once an intraneural site was located in which spontaneous bursts of MSNA could be recorded, neural activity was amplified 100 times via an MR‐compatible head‐stage located close to the recording site, further amplified to a total gain of 20,000 times and a bandpass of 300 Hz to 5 kHz (NeuroAmp EX; ADInstruments, Sydney, Australia), and stored on a computer using a computer‐based data acquisition and analysis system (PowerLab 16SP hardware and LabChart 7 software; ADInstruments, Sydney, Australia). Five minutes of continuous MSNA, BP (radial arterial tonometry; Colin 7000 NIBP; Colin Corp., Aichi, Japan), respiration (strain‐gauge transducer: Pneumotrace, UFI, Morro Bay, CA, USA), and HR (piezoelectric pulse transducer placed on the big toe) was then recorded with the subject relaxed. The BP monitor was then removed and the subject wheeled from the laboratory to the MRI facility.

### 2.3 MRI scanning and stimulus

Using a 3 Tesla MRI scanner (Philips Achieva, 32‐channel SENSE head coil), two scans encompassing the whole brain were collected: a high‐resolution 3D T1‐weighted anatomical image (200 axial slices, echo time [TE] = 2.5 ms, repetition time [TR] = 5,600 ms, raw voxel size = .87 mm^3^), followed by a series of 250 gradient echo echo‐planar blood oxygen level‐dependent functional magnetic resonance images (fMRI: 46 axial slices, TE = 40 ms, TR = 8 s, raw voxel size = 1.5 × 1.5 × 3.25 mm^3^). All 46 axial slices in each of the 250 fMRI brain volumes were collected during the first 4 s of the 8 s TR, which allowed for 4 s of clean MSNA recording per brain volume, that is, without the potential for scanner artifact.

Prior to the MRI scanning, two 10 ml syringes filled with 7% hypertonic saline were connected via a 30 cm length of extension tubing, primed with hypertonic saline, to a 23G butterfly cannula. The cannula was inserted 1.5 cm deep into the belly of the right tibialis anterior muscle, approximately 5 cm lateral and 10 cm inferior to the tibial tuberosity. An infusion of hypertonic saline was commenced during the 50th volume of the fMRI scan at the rate of .25 ml/min using an infusion pump (Harvard Instruments, USA), and this rate was constantly adjusted to maintain a pain level of 5 of 10 on a Numerical Rating Scale (0 = no pain, 10 = most intense pain imaginable). Subjects knew they were going to experience pain, but they were not informed as to the start time of the infusion. Throughout the entire scanning period, subjects gave feedback about their pain level by pressing four color‐coded buttons; each color was associated with either “pain onset,” “pain at 5/10,” “pain below 5/10,” “pain above 5/10.” At the completion of the scanning session, each subject was asked to complete a McGill Pain Questionnaire (Melzack, 1975) and draw the area of perceived pain on a standard diagram of the leg. This was then placed into ImageJ to determine the total area of spread (reported in number of pixels).

### 2.4 Analysis

#### 2.4.1 Muscle sympathetic nerve activity

**Figure 1:**
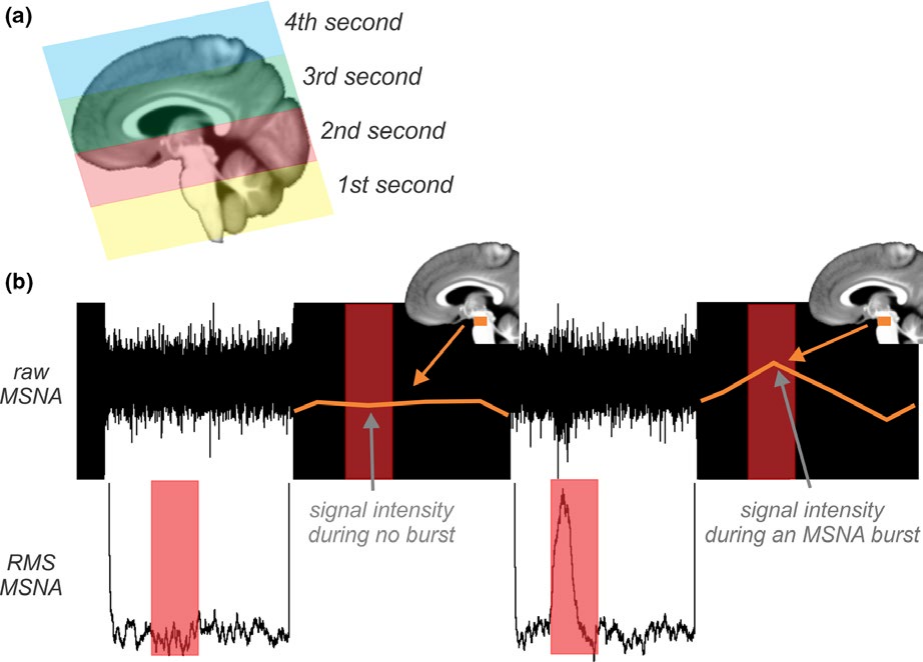
(a) Sagittal section of the brain showing the location of functional magnetic resonance imaging (fMRI) scans collected during the 4 s period. Images were collected in a caudal to rostral direction, enabling the analysis to target specific brain regions by only using the input of MSNA that occurred in the 1st (yellow), 2nd (red), 3rd (green), and 4th (blue) second of the 4 s interscan interval. (b) Recording of MSNA in a subject while performing fMRI of the brain. The filtered neurogram is shown in the top trace; the root‐mean‐square processed signal in the bottom trace. The black areas represent scanning artifacts during the 4 s “ON” periods when fMRI images were being acquired. The red shading corresponds to the 2nd second of the interscan interval. The signal intensity values for the highlighted brain region (orange rectangle) are schematically plotted as the orange line. The MSNA‐coupled signal intensity change was calculated for images in each second by averaging the signal during an MSNA burst and subtracting the signal during period of no bursts. MSNA, muscle sympathetic nerve activity; RMS, root‐mean‐square

**Figure 2:**
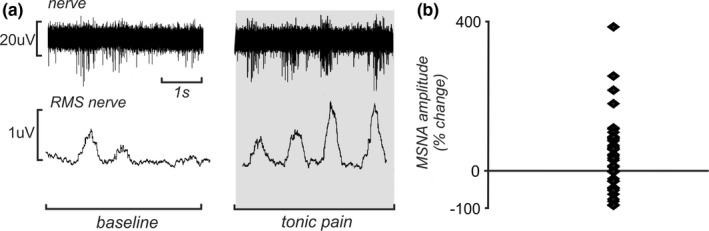
(a) Extract of an MSNA recording from the common peroneal nerve in a subject in whom MSNA amplitude increased during intramuscular infusion of hypertonic saline into the tibialis anterior muscle (gray shading, right), compared to baseline (left). Gray shading indicates the tonic pain period. (b) Plot of the mean percentage change in MSNA amplitude for the last 70 volumes of pain compared to baseline in 37 subjects. MSNA, muscle sympathetic nerve activity; RMS, root‐mean‐square; μV, microvolts

Individual bursts of MSNA were displayed as a mean voltage neurogram, computed as the root‐mean‐square (RMS) processed signal with a moving time average window of 200 ms. The 4 s interscan OFF period (see below) allowed the measurement of MSNA bursts. This 4 s period was divided into 1 s increments; for each second, bursts were manually counted, and the amplitude was measured from the RMS‐processed nerve signal (Figures 1b and 2a).

#### 2.4.2 Brain image processing

Using SPM12 (Friston et al., 1994) and custom software, all fMRI images were realigned and coregistered to each individual's T1‐weighted image set. The T1 image was then spatially normalized to the Montreal Neurological Institute (MNI) template, and the normalization parameters applied to all fMRI images. We used the VBM8 toolbox DARTEL template. This template was derived from 550 healthy control subjects of the IXI database (http://brain-development.org/) and is in MNI152 space. In all subjects, no pain was reported to occur during the first 70 brain volumes (or 9.33 min) and thus we selected this period to be the *baseline period*. In contrast, we found that between volumes 181 and 250, subjects reported sustained pain, and there was a significant change in MSNA; we selected this 70‐volume (9.33 min) period as the *tonic pain period*. These periods were selected and separated so that they could then be detrended separately, in order to remove the potential influence of sustained changes in overall intensity within each voxel. The 70 volumes in each baseline and tonic pain period were then linearly detrended, bias‐corrected, and spatially smoothed using a 6 mm full‐width at half‐maximum (FWHM) Gaussian filter. In addition, we performed a brainstem‐specific analysis in subjects for whom we acquired images that extended to the caudal medulla (*n* = 30). In each subject, the brainstem was isolated from the T1‐weighted anatomical image and the parameters applied to the coregistered, realigned, and detrended fMRI images. The T1 images set was normalized to a brainstem template in MNI space using the SUIT toolbox and the parameters applied to the fMRI images sets (Diedrichsen et al., 2011). These fMRI brainstem image sets were then spatially smoothed using a 4 mm FWHM Gaussian filter.

As mentioned above, during the fMRI scan, all axial slices were collected during the first 4 s of the 8 s TR. Signal intensity changes were measured during the subsequent 4 s (ON period), taking into account the (+) 5 s neurovascular coupling delay between a neuronal event and the peak BOLD signal (Logothetis, Pauls, Augath, Trinath, & Oeltermann, 2001), and the (−) 1 s required for the burst of MSNA to travel from the brain to the peripheral recording site (Fagius & Wallin, 1980). Because the images were collected in a caudal to rostral direction, the region of the brainstem from the caudal medulla to the rostral pons was scanned in the 1st second, the rostral pons to the diencephalon in the 2nd second, the diencephalon and surrounding cortex in the 3rd second, and the remainder of the cortex in the 4th second (Figure 1a).

In each subject, and for each of the 4 s periods, the brain volumes during periods in which there were no bursts were averaged to create a mean *no MSNA burst* image. Similarly, brain volumes during periods in which there were MSNA bursts were averaged to create a mean *MSNA burst* image. These two images were created for the baseline and tonic pain periods separately (Figure 1b). To determine brain areas in which signal intensity was greater during MSNA bursts compared to periods of no MSNA bursts, we entered these two images for the baseline period into a second level, random‐effects paired *t* test for each of the 4‐s periods. An initial threshold of *p* < .001, uncorrected was used to display regional changes. We then performed cluster correction (family‐wise error *p* < .05) to limit the prospects of Type II errors (Woo, Krishnan, & Wager, 2014). Clusters were overlaid onto a mean whole‐brain and brainstem T1‐weighted anatomical image calculated from all subjects in the study.

In addition, to determine changes in MSNA‐coupled BOLD signal intensity evoked by tonic pain, we subtracted the mean *MSNA no burst* image from the mean *MSNA burst* image during the baseline and tonic pain periods. This resulted in a single baseline and a single tonic pain image for each subject, of which each voxel's signal intensity value reflected the difference between periods of no bursts compared to periods of MSNA bursts. The percentage difference between these two images was then calculated for each voxel, resulting in a brain map in which each voxel's value was the percentage change in MSNA‐coupled signal intensity during tonic pain compared with baseline. These brain maps were placed into a second‐level random‐effects analysis, where relationships between percentage change in MSNA‐coupled signal intensity and percentage change in MSNA amplitude during tonic pain were determined (*p* < .001, uncorrected). Significant clusters were overlaid onto a mean whole‐brain and brainstem T1‐weighted anatomical image, calculated from all subjects in the study. Finally, for each significant cluster, the percentage differences in MSNA‐coupled signal intensity changes during pain compared with baseline were extracted and plotted against the overall change in MSNA amplitude during pain.

## 3 RESULTS

### 3.1 Participants

The resting mean BP was 86.3 ± 2.0 mmHg (range: 68.7–105.5 mmHg), resting mean HR was 66.4 ± 1.7 beats/min (range: 50–85 beats/min), resting MSNA frequency was 12.0 ± 1.5 bursts/min (range: 2–35 bursts/min), MSNA amplitude change for the last pain period was +42.7 ± 16.6% (range: −91.7 to +388.1%), PCS: 12.0 ± 1.2 (range: 0–27), PASS: 28.8 ± 2.8 (range: 2–72), PVAQ: 34.7 ± 1.6 (range: 16–49), state anxiety: 31.4 ± 1.9 (range: 20–52), and trait anxiety: 35.1 ± 1.4 (range: 24–50); see Table 1.

**Table 1:** Mean (±*SEM*) physiological and psychometric values for all subjects

	Sex	Age	Mean BP (mmHg)	HR (beats/min)	Resting MSNA (bursts/min)	MSNA amplitude during pain (% change)	PCS	PASS	PVAQ	State anxiety	Trait anxiety
Mean	11 F, 26 M	22.0	86.3	66.4	14.3	42.7	12.0	28.8	34.7	31.4	35.1
*SEM*		0.5	2.0	1.7	1.7	16.6	1.2	2.8	1.6	1.9	1.4

F, female; M, male; BP, blood pressure; HR, heart rate; MSNA, muscle sympathetic nerve activity; PASS, Pain Anxiety Symptoms Scale; PCS, Pain Catastrophizing Scale; PVAQ, Pain Vigilance and Awareness Questionnaire.

### 3.2 Psychophysics

In all subjects, intramuscular infusion of hypertonic saline elicited continuous muscle pain, which began, on average, 22 volumes (176 s) after the start of the infusion (i.e., at volume 72) and continued at a mean pain intensity of 5.8 ± .1 (range: 5–7) for the remainder of the fMRI scan. The mean volume of infusion was 18.6 ± 1.4 ml. The mean pain spread depicted on the McGill pain questionnaire involved an area of 1,039 ± 80 pixels (as measured from the area of pain subjects drew on an image of a leg). The most frequent descriptors chosen from the McGill pain questionnaire to describe the tonic pain were “dull,” “aching,” and “throbbing.” The results from the PCS, PVAQ, PASS, and STAI questionnaires were all within normal range. A score ≥30 in the PCS represents a clinically significant level of catastrophizing attitude toward pain (Sullivan et al., 1995). Scores higher than 39–40 in the STAI suggest clinically significant symptoms (Knight, Waal‐Manning, & Spears, 1983; Spielberger et al., 1983). PVAQ results can range between 0 and 80 (McCracken, 1997), and the PASS‐20 between 0 and 100 (McCracken & Dhingra, 2002) (Table 1).

### 3.3 MSNA rest versus pain

The mean percentage change during the last 70 volumes of pain compared to the 70 volumes of baseline was +42.7 ± 16.6%. The amplitude change during this period ranged between −91.7% and +388.1% across the 37 subjects (Figure 2b).

### 3.4 MSNA‐coupled signal intensity changes

#### 3.4.1 Baseline

**Figure 3:**
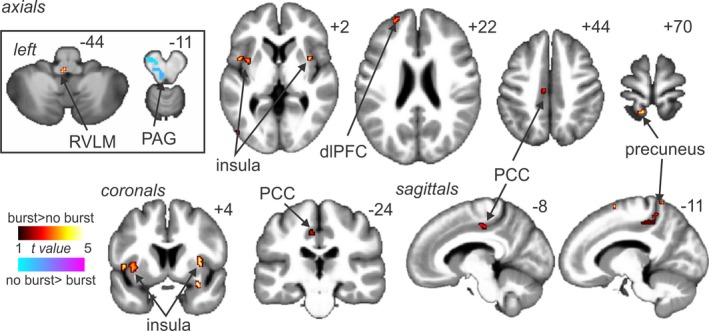
Signal intensity increases (hot color scale) and decreases (cool color scale) during MSNA bursts compared to periods of no bursts, at rest in all 37 subjects. Significant clusters were overlaid onto a mean T1‐weighted anatomical image set created from all 37 subjects. Slice locations in Montreal Neurological Institute space are indicated in the top right of each image. The left side is the side contralateral to the side of microneurography recording. The inset on the left represents the results from a brainstem‐specific analysis. MSNA, muscle sympathetic nerve activity; dlPFC, dorsolateral prefrontal cortex; PAG, periaqueductal gray; PCC, posterior cingulate cortex; RVLM, rostral ventrolateral medulla

During the baseline period, increases in MSNA‐coupled signal intensity occurred in the rostral medulla encompassing the area of the left rostral ventrolateral medulla (RVLM; mean ± *SEM* percent change burst vs. no burst: .34 ± .14), left midinsula (.17 ± .03), right midinsula (.17 ± .05), left posterior cingulate cortex (PCC; .14 ± .04), left dorsolateral prefrontal cortex (dlPFC; .22 ± .07), and left precuneus (.27 ± .07) (Figure 3, Table 2). In contrast, decreases in MSNA‐coupled signal intensity occurred in only one region, in the rostral midbrain encompassing the region of the left midbrain PAG (−.25 ± .07).

**Table 2:** Locations in Montreal Neurological Institute space, *t* values, and cluster sizes of regions in which signal intensity increased or decreased during muscle sympathetic nerve activity bursts compared with periods of no MSNA bursts, while the subject was at rest

	*x*	*y*	*z*	*t* value	Cluster size
*Signal intensity increases*					
Dorsolateral prefrontal cortex					
Left	−22	54	24	3.41	6
Insular cortex					
Left	−34	2	0	5.07	65
Right	38	6	2	3.62	15
Posterior cingulate cortex					
Left	−6	−22	44	3.84	13
Precuneus					
Left	−12	−52	70	3.55	7
Rostral ventrolateral medulla					
Left	−2	−40	−43	2.58	4
*Signal intensity decreases*					
Periaqueductal gray					
Left	−8	−26	−11	2.84	18

MSNA, muscle sympathetic nerve activity.

#### 3.4.2 Tonic pain

**Figure 4:**
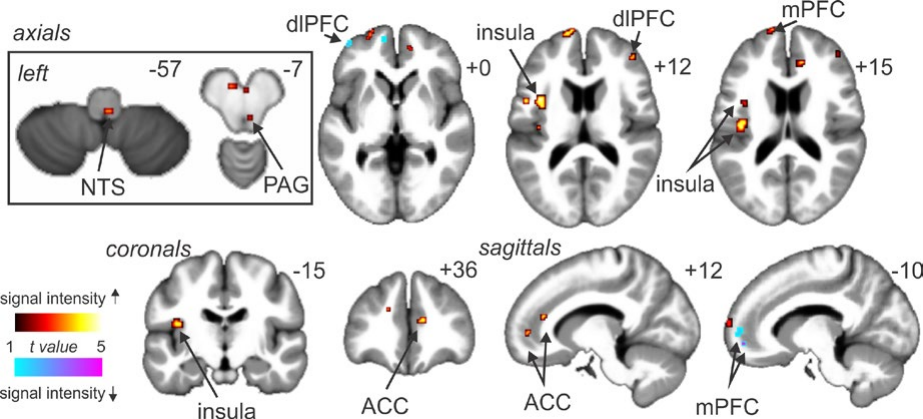
Brain regions in which muscle sympathetic nerve activity (MSNA)‐coupled signal intensity changes were significantly positively (hot color scale) or negatively (cool color scale) correlated with MSNA amplitude change in 37 subjects. Significant clusters were overlaid onto a mean T1‐weighted anatomical image set created from all 37 subjects. Slice locations in Montreal Neurological Institute space are indicated in the top right of each image. The inset on the left represents the results from a brainstem‐specific analysis. ACC, anterior cingulate cortex; dlPFC, dorsolateral prefrontal cortex; mPFC, medial prefrontal cortex; NTS, nucleus tractus solitarius; PAG, periaqueductal gray

**Figure 5:**
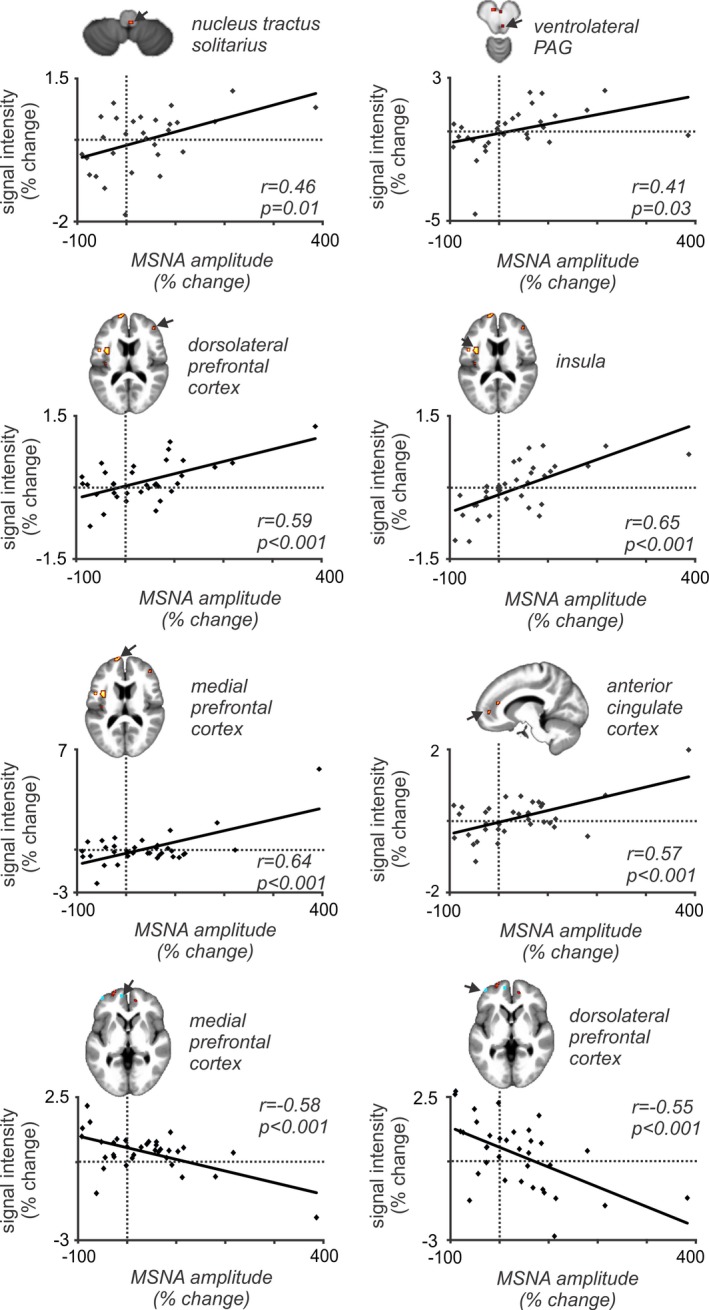
Plots of the muscle sympathetic nerve activity (MSNA)‐coupled signal intensity changes against MSNA amplitude change during tonic pain in all 37 subjects. In all regions, there is a significant linear relationship between MSNA burst‐to‐burst signal changes during tonic pain compared with baseline with the overall change in MSNA amplitude. PAG, periaqueductal gray

During tonic pain, a number of brain regions displayed significant correlations between percentage changes in MSNA‐coupled signal intensity and MSNA amplitude (Figures 4 and 5, Table 3). It can be seen in Figure 5 that one subject had a very high MSNA value, accordingly we have performed statistical analyses with and without the outlier. Significant positive relationships occurred in the dorsal closed medulla in the region of the right nucleus tractus solitarius (NTS; *r* = .46, *p* = .01; *r* = .43, *p* = .02 without outlier), right midbrain in the region of the ventrolateral PAG (*r* = .41, *p* = .03; *r* = .57, *p* = .001 without outlier), left insula (*r* = .65, *p* < .001; *r* = .48, *p* = .004 without outlier), left medial prefrontal cortex (mPFC; *r* = .64, *p* < .001; *r* = .28, *p* = .10 without outlier), right dlPFC (*r* = .59, *p* < .001; *r* = .42 *p* = .01 without outlier), and the right anterior cingulate cortex (ACC; *r* = .57, *p* < .001; *r* = .28, *p* = .11 without outlier). Significant negative relationships occurred in the left dlPFC (*r* = −.55, *p* < .001; *r* = −.55, *p* < .001 without outlier) and the left mPFC (*r* = −.58, *p* < .001; *r* = −.32, *p* = .06 without outlier). After excluding the outlier, significant positive relationships were still apparent for the NTS, PAG, left insula, and significant negative relationships for the right dlPFC and left mPFC.

**Table 3:** Locations in Montreal Neurological Institute space, *t* values, and cluster sizes of regions in which MSNA‐coupled signal intensity changes during tonic muscle pain were significantly different to those at rest

	*x*	*y*	*z*	*t* value	Cluster size
*Signal intensity increases*					
Anterior cingulate cortex					
Right	15	50	0	3.74	3
	10	34	16	3.81	4
Dorsolateral prefrontal cortex					
Right	38	36	14	3.64	4
Insular cortex					
Left	−40	−18	14	4.05	37
	−36	6	12	4.07	27
Medial prefrontal cortex					
Left	−6	64	8	5.04	45
Midbrain periaqueductal gray					
Right	4	−32	−7	2.33	1
Nucleus tractus solitarius					
Right	2	−44	−57	2.66	2
*Signal intensity decreases*					
Dorsolateral prefrontal cortex					
Left	−40	52	2	4.21	5
Medial prefrontal cortex					
Left	−8	56	4	4.07	27

MSNA, muscle sympathetic nerve activity.

## 4 DISCUSSION

In this study, we reveal MSNA‐coupled signal intensity changes produced by tonic muscle pain. Although many previous investigations have explored changes in signal intensity associated with changes in overall MSNA during various challenges, this is the first to compare signal intensity changes during MSNA bursts at baseline and during a challenge such as tonic muscle pain. We found that during tonic pain MSNA‐coupled signal intensity changes were positively correlated to MSNA amplitude in areas of the brainstem known to regulate the cardiovascular responses to pain, such as the PAG. In addition, we found both positive and negative correlations between MSNA‐coupled signal changes and MSNA amplitude in higher order association areas, such as the insular, cingulate, and prefrontal cortices. Some of these regions also showed MSNA‐coupled signal changes at rest, as we had shown previously (James, Macefield, & Henderson, 2013; Lundblad et al., 2014), indicating that they are modulated during tonic pain to evoke an overall change in MSNA.

Hypertonic saline infusion into the tibialis anterior muscle induced pain that was described as dull, aching, and throbbing. Consistent with our previous investigations, we found that tonic muscle pain evoked increases in MSNA amplitude in some individuals and decreases in others (Fazalbhoy et al., 2012, 2014; Kobuch et al., 2015, 2016, 2017). These changes began approximately 20 volumes (160 s) after the start of the hypertonic infusion, at approximately the same time as subjects began to perceive pain, and remained fairly stable for the duration of the pain period. Importantly, and unlike any previous investigation, we have determined brain regions that influence MSNA amplitude in a *burst‐to‐burst* (MSNA‐coupled) manner rather than in a *sustained* manner during a cardiovascular challenge. Previous studies investigating brain activation patterns during various cardiovascular challenges have explored gradual signal intensity changes associated with sustained changes in BP, HR, MSNA, or other autonomic variables (Beissner, Meissner, Bär, & Napadow, 2013 for review; Harper et al., 2003 [cold pressor]; Henderson et al., 2002 [Vasalva]). Although these studies are valuable, they are unable to explore signal intensity changes in brain regions that directly drive each MSNA burst. We find in this study a series of brain regions that are only revealed through such an analysis, which provides a basis for exploring regions that may be recruited only during various cardiovascular challenges and only in a burst‐to‐burst manner.

Consistent with our previous studies, we found that at rest, signal intensity changes coupled to MSNA bursts occurred in the region of the RVLM, insula, cingulate, prefrontal cortex, and precuneus (James et al., 2013; Lundblad et al., 2014; Macefield & Henderson, 2010). These results were consistent despite a subtle difference in the analysis methods, that is, signal intensity changes coupled to MSNA burst *pattern*, versus mean signal during MSNA bursts compared with no bursts. One region that was different between this and our previous investigations was the PAG; we previously reported positive signal coupling with MSNA, whereas in this study, we found decreased MSNA‐coupled signal intensity in the PAG at rest (Lundblad et al., 2014). Why this disparity occurs is not clear; however, it is possible that it arises from differences in methodological approaches. In addition to the PAG, tonic pain evoked MSNA‐coupled signal changes associated with MSNA amplitude in other regions, which also display MSNA‐coupled signal changes at rest, such as the NTS, and the insular, cingulate, and prefrontal cortices. This suggests that these regions are not necessarily recruited during tonic pain itself but instead are modulated so that their activities drive either increases or decreases in MSNA amplitude.

Consistent with this idea, we found that the PAG, the right dlPFC, and the right ACC each displayed both burst‐to‐burst *and* sustained signal intensity changes during tonic muscle pain (Kobuch et al., 2017). Indeed, we previously found that the dorsolateral PAG (dlPAG) displayed *sustained* increases in signal intensity during tonic pain that were negatively correlated to MSNA amplitude, that is, the greater the signal change the lower the MSNA amplitude change (Kobuch et al., 2017). In contrast, in the current study, we found that positively correlated MSNA‐coupled signal changes during muscle pain occurred in the region of the ventrolateral PAG (vlPAG).

The PAG is arranged in a columnar fashion, based on histological and functional differences (Keay & Bandler, 2001). In animals, it is well established that the PAG is activated by both superficial and deep pain. Indeed, superficial pain activates the lateral PAG column, and direct stimulation of this column of the PAG elicits active behavioral responses that are coupled with increases in BP, HR, and sympathetic activity (Bandler et al., 2000; Carrive, Dampney, & Bandler, 1987, 1988; Hilton & Redfern, 1986; Meller & Dennis, 1991; Van Bockstaele, Aston‐Jones, Pieribone, Ennis, & Shipley, 1991; Yardley & Hilton, 1986). In contrast, deep pain activates the ventrolateral column of the PAG, and direct stimulation of this column evokes passive, quiescent coping behaviors, and decreases in BP, HR, and sympathetic activity (Carrive & Bandler, 1991; Lovick, 1992). Further, unlike the vlPAG, which receives noxious muscle and visceral inputs from the spinal cord and brainstem, the dlPAG receives inputs from primarily prefrontal and cingulate cortices (Floyd, Price, Ferry, Keay, & Bandler, 2000). This difference in input patterns has led to the suggestion that the dlPAG produces active coping strategies in response to psychological stressors, whereas the vlPAG produces passive coping responses in response to deep physical stressors (Keay & Henderson, 2010). This idea is consistent with our own findings; although overall signal intensity within the vlPAG did not change during tonic muscle pain, MSNA‐coupled signal intensity changed in a manner consistent with each individual's overall MSNA response. Increases in MSNA‐coupled signal intensity in the vlPAG paralleled increases in MSNA amplitude, and MSNA‐coupled signal intensity decreases in the vlPAG were accompanied by decreases in MSNA amplitude. It is possible that the vlPAG is involved in driving the change in MSNA burst amplitude on a burst‐to‐burst basis, whereas the dlPAG may provide more of a tonic modulatory role during tonic muscle pain.

Similar to the PAG, the ACC and dlPFC displayed MSNA‐coupled signal intensity changes, which paralleled MSNA amplitude changes. Increases in MSNA amplitude were accompanied by increases in MSNA‐coupled signal intensity in these regions, and decreases in MSNA amplitude were accompanied by decreases in MSNA‐coupled signal intensity in these regions. Further, we previously showed that these two regions displayed sustained intensity increases during tonic muscle pain in a pattern that matched the increase in MSNA amplitude (Kobuch et al., 2017). A role for these regions in mediating changes in autonomic function has been recognized for some time. For example, the ACC and dlPFC are both activated during cardiovascular challenges such an inspiratory capacity apnea (Kimmerly, Morris, & Floras, 2013; Macefield, Gandevia, & Henderson, 2006). Furthermore, we have shown that these regions display MSNA‐coupled signal changes even at rest.

In our previous study, we found that the insular cortex did not display sustained signal intensity changes coupled to sustained changes in MSNA during tonic muscle pain (Kobuch et al., 2017). This is surprising given the growing body of the literature describing insular cortex changes during various cardiovascular challenges (Beissner et al., 2013; Butcher & Cechetto, 1995; Harper et al., 2003; Henderson et al., 2002; Macefield et al., 2006; Macey et al., 2003). However, we found in the current study that the left insula displayed robust changes in MSNA‐coupled signal intensity that were indeed associated with overall changes in MSNA. Consistent with previous findings, the left and right insula exhibited MSNA‐coupled signal intensity increases at rest (Fatouleh et al., 2014; James et al., 2013). During tonic pain, this MSNA‐coupled signal change was located more dorsally in mid‐ and anterior insula and only on the left side, contralateral to the noxious stimulus. We have previously reported that intramuscular injection of hypertonic saline into the right tibialis anterior muscle causes sustained signal increases in the contralateral posterior insula and ipsilateral anterior insula and not in the contralateral anterior insula (Henderson, Gandevia, & Macefield, 2007). It has been proposed that these regions are involved in the link between sensory and emotional aspects of pain processing (Craig, 2011). The data presented here reveal that the contralateral mid‐ and anterior dorsal insula display MSNA‐coupled signal changes during pain and are thus likely involved in mediating the cardiovascular aspect of pain. Interestingly, it has been revealed that the mid‐ and anterior insula are activated during a range of autonomic challenges such as the Valsalva maneuver, handgrip, and cold pressor test, whereas the posterior insula is not (Macey et al., 2012). This raises the prospect that different insula regions may be preferentially involved in mediating the sensory, affective, and cardiovascular response to sustained muscle pain.

In direct contrast to the above‐mentioned regions, in which the magnitudes of MSNA‐coupled signal changes alter during tonic muscle pain, a number of important brain regions did not display pain‐related changes. For example, although we found that the RVLM and precuneus displayed MSNA‐coupled signal changes at rest, consistent with our earlier studies (James et al., 2013; Macefield & Henderson, 2010), these signal intensity increases did not change in magnitude during each burst of MSNA during tonic muscle pain. However, both of these regions displayed *sustained* signal intensity increases in the increasing MSNA group and decreases in the decreasing MSNA group (Kobuch et al., 2017). Therefore, the RVLM and precuneus are not recruited to fire either more or less in a burst‐to‐burst fashion during tonic muscle pain but instead may provide a tonic modulatory role.

Finally, it is important to note that the areas identified in this study overlap with regions thought to be involved in descending pain modulation (Millan, 2002). Furthermore, it is interesting that there appears to be a relationship between the cardiovascular and descending pain modulation systems, as the greater the magnitude of cardiovascular response during the cold pressor test, the greater an individual's endogenous analgesic ability (Chalaye, Devoize, Lafrenaye, Dallel, & Marchand, 2013). Our findings that MSNA‐related regions overlap with those involved in endogenous analgesia further support the idea that the two systems are intertwined.

### 4.1 Limitations

As with all studies, there are limitations to the technique and methods of analysis. While the brainstem is small relative to the spatial resolution obtained in most fMRI investigations, one of the major limitations of brainstem analysis is accurate spatial normalization. To overcome this issue, we used a brainstem‐specific toolbox, which isolates the brainstem; we then manually selected the brainstem in each individual subject. This was then used to spatially normalize the image sets, which creates greater consistency between subjects with respect to their brainstems’ final location in MNI space. Given this, we are confident that the changes we report in this study, particularly those in the brainstem, are accurate. Another limitation relates to the number of volumes chosen for the baseline and tonic pain periods. We collected 70 brain volumes prior to the onset of pain and, to be consistent, we chose 70 volumes during the tonic pain period, when the level of pain was stable. A greater number of volumes in both periods would have been desirable, to increase reliability, and in future investigations, we aim to collect longer baseline periods. We did, however, find consistent MSNA‐coupled signal changes at rest when compared to our previous studies in which we analyzed 200 brain volumes, so we are confident that the results presented are indeed accurate.

Finally, we acknowledge that the regional hemodynamic response curve has a wide range and therefore using a fixed 4‐s ON and 4‐s OFF protocol prevents us from taking into account individual variations. However, we have used this same protocol in many previous investigations where we concurrently recorded MSNA and fMRI and have demonstrated that the BOLD signal intensity is temporally coupled to the bursts of MSNA recorded peripherally. Indeed, MSNA‐coupled fMRI allows one to identify regions that are temporally coupled to the firing of an MSNA burst, which (with the exception of certain pathophysiological states) do not occur in every heartbeat. In our previous investigations, we had shown that both increases and *decreases* in BOLD signal occurred. Importantly, these were not *general* changes in BOLD intensity, which one would expect from physiological noise related to ongoing cardiac (and respiratory) pulsations within the brain, but discrete. In this study, we were not able to filter out potential physiological noise (see Brooks, Faull, Pattinson, & Jenkinson, 2013) as we were exploring MSNA‐coupled signal intensity changes, and MSNA bursts are tightly coupled to the cardiac rhythm. Removing frequencies (or their harmonics) that represent cardiac pulsatile signals would also remove MSNA‐coupled signal changes. As periods in which there were no MSNA bursts also contain cardiac beats, any potential effect of physiological noise would be equivalent during both periods. Furthermore, given that the discrete areas of increase or decrease in BOLD signal were localized to cortical and subcortical regions known to contribute to cardiovascular regulation in experimental animals, the most parsimonious explanation is that the observed changes in BOLD signal intensity reflect proxy markers of functional changes in neuronal activity, rather than physiological artifact.

## 5 CONCLUSIONS

For the first time, we have shown brain regions in which MSNA‐coupled signal intensity changes are altered during a challenge that affects sympathetic outflow to the muscle vascular bed. While it is important to understand brain regions that are associated with sustained changes in sympathetic drive, we show here that signal intensity changes in numerous brain regions occur only in a burst‐to‐burst manner. We provide evidence that some regions display both sustained and burst‐to‐burst changes, whereas others display one or the other. These data are consistent with the idea that changes in MSNA result from a combination of sustained and burst‐to‐burst changes in activity in various brain regions. Whether these changes persist during chronic pain remains unknown. However, investigating these potential changes in chronic pain would require knowing the baseline parameters prior to the development of chronic pain.

## CONFLICT OF INTERESTS

The authors declare no conflict of interests, financial, or otherwise.
